# Connection Input Mapping and 3D Reconstruction of the Brainstem and Spinal Cord Projections to the CSF-Contacting Nucleus

**DOI:** 10.3389/fncir.2020.00011

**Published:** 2020-03-31

**Authors:** Si-Yuan Song, Ying Li, Xiao-Meng Zhai, Yue-Hao Li, Cheng-Yi Bao, Cheng-Jing Shan, Jia Hong, Jun-Li Cao, Li-Cai Zhang

**Affiliations:** Jiangsu Province Key Laboratory of Anesthesiology, Xuzhou Medical University, Xuzhou, China

**Keywords:** CSF-contacting nucleus, brainstem, spinal cord, projection, retrograde trace

## Abstract

**Objective:**

To investigate whether the CSF-contacting nucleus receives brainstem and spinal cord projections and to understand the functional significance of these connections.

**Methods:**

The retrograde tracer cholera toxin B subunit (CB) was injected into the CSF-contacting nucleus in Sprague-Dawley rats according the previously reported stereotaxic coordinates. After 7–10 days, these rats were perfused and their brainstem and spinal cord were sliced (thickness, 40 μm) using a freezing microtome. All the sections were subjected to CB immunofluorescence staining. The distribution of CB-positive neuron in different brainstem and spinal cord areas was observed under fluorescence microscope.

**Results:**

The retrograde labeled CB-positive neurons were found in the midbrain, pons, medulla oblongata, and spinal cord. Four functional areas including one hundred and twelve sub-regions have projections to the CSF-contacting nucleus. However, the density of CB-positive neuron distribution ranged from sparse to dense.

**Conclusion:**

Based on the connectivity patterns of the CSF-contacting nucleus receives anatomical inputs from the brainstem and spinal cord, we preliminarily conclude and summarize that the CSF-contacting nucleus participates in pain, visceral activity, sleep and arousal, emotion, and drug addiction. The present study firstly illustrates the broad projections of the CSF-contacting nucleus from the brainstem and spinal cord, which implies the complicated functions of the nucleus especially for the unique roles of coordination in neural and body fluids regulation.

## Introduction

The cerebrospinal fluid (CSF)-contacting nucleus is a unique nucleus in the brain. It is located within the ventral gray of the lower portion of the aqueduct (Aq) and upper portion of the fourth ventricle (4V) floor (Song et al., 2019). The outstanding feature of this nucleus is that the neural somata are located in the brain parenchyma with their axons stretching into the CSF (Song and Zhang, 2018; Song et al., 2019). The morphological connections of CSF-contacting nucleus with non-CSF-contacting neurons, glial cells, and blood vessels have been confirmed using electron microscopy (Zhang et al., 2003). The unique characteristic feature of the CSF-contacting nucleus is that it may be a key structure bridging the nerves and fluids (CSF and plasma). The study of this nucleus is about 30 years after we discovered and named this nucleus. The basic biological characteristics of the CSF-contacting nucleus such as specific labeling method (Lu et al., 2008), location and stereotaxic coordinates (Song et al., 2019), receptor, neurotransmitter, and ion channel distributions (Lu et al., 2011; Wang et al., 2014; Liu et al., 2017), and their relationships with morphine dependence and withdrawal, stress, sodium appetite, and pain (Lu et al., 2011; Wu et al., 2015; Xing et al., 2015; Zhou et al., 2017) have been revealed. However, the pathways and mechanisms associated with such biological activities have not been elucidated yet.

The CSF-contacting nucleus is also located in the brainstem and participates in pain and other behaviors (Lu et al., 2011; Xing et al., 2015; Liu et al., 2017). To demonstrate the functions and neural networks of the CSF-contacting nucleus in the central nervous system, it is import to identify the existence of connections from the brainstem and spinal cord to the CSF-contacting nucleus and to elucidate the connections from different functional regions. Therefore, we planned to inject retrograde tracer cholera toxin B subunit (CB) into the CSF-contacting nucleus. By using immunofluorescence technique, the projections from the brainstem and spinal cord to the CSF-contacting nucleus can be revealed. The possible functional significance can be speculated according to the projection relationships, which will deepen the understanding and lay a foundation for future research.

## Materials and Methods

### Experimental Animals

Specific pathogen-free (SPF) grade Sprague-Dawley rats weighing 250 ± 50 g were acquired from the Experimental Animal Center of Xuzhou Medical University. Rats successfully injected with the tracer into the CSF-contacting nucleus were used for observation and analysis (*n* = 6). All the animals were housed under a 12/12 light/dark cycle at the temperature of 22–25°C, with *ad libitum* access to food and water.

### Tracer Administration

Rats were anesthetized with pentobarbital sodium (40 mg/kg, i.p.), and their heads were fixed on the stereotaxic instrument (Stoelting 51700, United States). Retrograde tracer CB solution (0.2 μl, 1%; Sigma, United States) was injected into the core of the CSF-contacting nucleus (Posterior to Bregma: 8.24 ± 0.18 mm, Lateral: 0.09 ± 0.01 mm, Depth: 6.45 ± 0.11 mm) (Song et al., 2019) by using Hamilton syringe with a 33G needle tip (Hamilton Company, Switzerland). The injection was applied under the microinfusion pump (KD Scientific, United States) for over 30 min periods. At the end of the injection, the microsyringe was left in place for 10–15 min before retraction.

### Sampling and Sectioning

After 7–10 days of retrograde tracer CB injections, rats were perfused transcardially and sacrificed. The whole brain and spinal cord were isolated and sectioned (40 μm thickness) coronally using a cryostat (Leica CM1900, Germany). All the sections were placed in sequence and numbered. In this study, only the brainstem and spinal cord were analyzed.

### Tracer Staining and CB-Positive Neuron Enumeration

After CB immunofluorescent staining (rabbit anti-CB primary antibody diluted in 1:600, Abcam; donkey anti-rabbit Alexa Fluor 488 secondary antibody diluted in 1:200, Life Technologies), the sections were sequentially mounted on slides, counterstained with DAPI, and coverslipped. The sections of the brainstem and spinal cord were visualized using fluorescence (Leica DM6, Germany) and confocal laser microscopes (Zeiss LSM 880, Germany) for different positions at the uniform standard. The cell density of CB-positive neurons (cell number/0.2 mm^2^ area) in each brain region was calculated using Image-Pro Plus 7.0 software. The density of CB-positive neurons was classified according to the densities: <5, 6–10, and >10, which correspond to sparse, moderate, and dense distributions, respectively.

### Verification of the Retrograde Results by Anterograde Tracing

The recombinant adenoassociated virus vector (serotype 2/9) expressed enhanced GFP (EGFP) under control of a human synapsin I promoter was used for anterograde tracing to verify the retrograde tracing results. 0.2 μl rAAV2/9-hSyn-EGFP (1.2 × 10^12^ gene copies; Brainvta Company, Wuhan, China) was injected into the raphe magnus nucleus (RMg) (Posterior to Bregma: 10.92 ± 0.2 mm, Lateral: 0 mm, Depth: 10.5 ± 0.1 mm) (Paxinos and Watson, 2007). 1 μl (1 μg) Cholera toxin subunit B Alexa Fluor 555 conjugate (CB-555) (Brainvta Company, Wuhan, China) was microinjected into the lateral ventricle (Posterior to Bregma: 1.0 ± 0.1 mm, Lateral: 1.6 ± 0.1 mm, Depth: 4.0 ± 0.2 mm) (Paxinos and Watson, 2007) to label the CSF-contacting nucleus. The injection parameters were the same as above. Rats were perfused after 14–21 days of anterograde tracer rAAV2/9 injections. The axon terminals from the RMg to the CSF-contacting nucleus were captured under confocal laser microscopes (Zeiss LSM 880, Germany).

### 3D Reconstruction of the Brainstem and Spinal Cord Connections

The CB-positive neurons were aligned, segmented, and registered into rat common reference atlas (Paxinos and Watson, 2007). The 3D connections were reconstructed by using Imaris software version 8.4.1 (Bitplane, United States).

### Statistics

SPSS 13.0 software was used for data analysis in the present study. Data are presented as mean ± SD.

## Results

### Injection of Retrograde Tracer Cholera Toxin B Subunit Into the CSF-Contacting Nucleus

**FIGURE 1:**
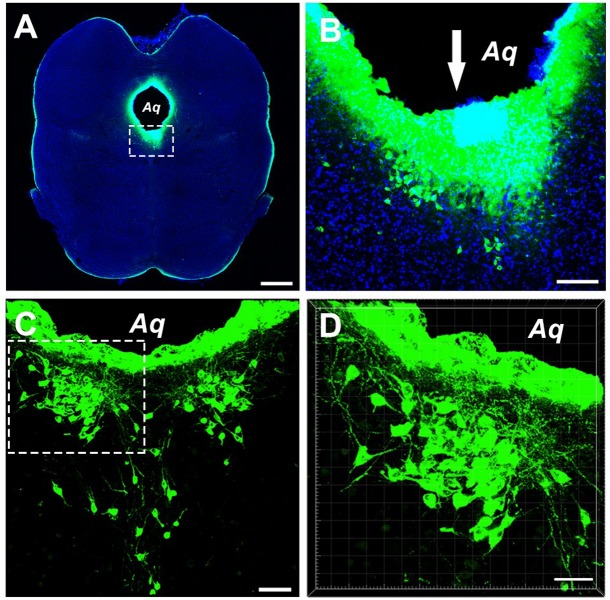
Photographs showing injection of the tracer CB into the CSF-contacting nucleus. **(A)** The site of injection of tracer CB into the CSF-contacting nucleus. Green fluorescence labeling of CB can be seen in the wholly CSF-contacting nucleus. **(B)** Higher magnification of the boxed area in **(A)**. Downwards arrow (↓) shows the passage of the injected needle. **(C)** The representative section of the CSF-contacting nucleus in the brain. **(D)** Higher magnification of the boxed area in **C**. Aq, aqueduct. Scale bar = 1 mm in **(A)**, 100 μm in **(B)**, 70 μm in **(C)**, and 40 μm in **(D)**.

Injections of the CB tracer produced dense immunofluorescence staining (green). The tracer was confined within the boundary of the CSF-contacting nucleus (Figures 1A,B), where the microsyringe needle tract can be seen to be located at the core of the CSF-contacting nucleus. Representative sections of the CSF-contacting nucleus are shown in Figures 1C,D.

### Cellular Morphology of Brainstem and Spinal Cord Connections

After the retrograde tracer was injected into the CSF-contacting nucleus, it was transported in a retrograde manner along the axons; consequently, the neural somata projected from the brainstem and spinal cord can be detected.

**FIGURE 2:**
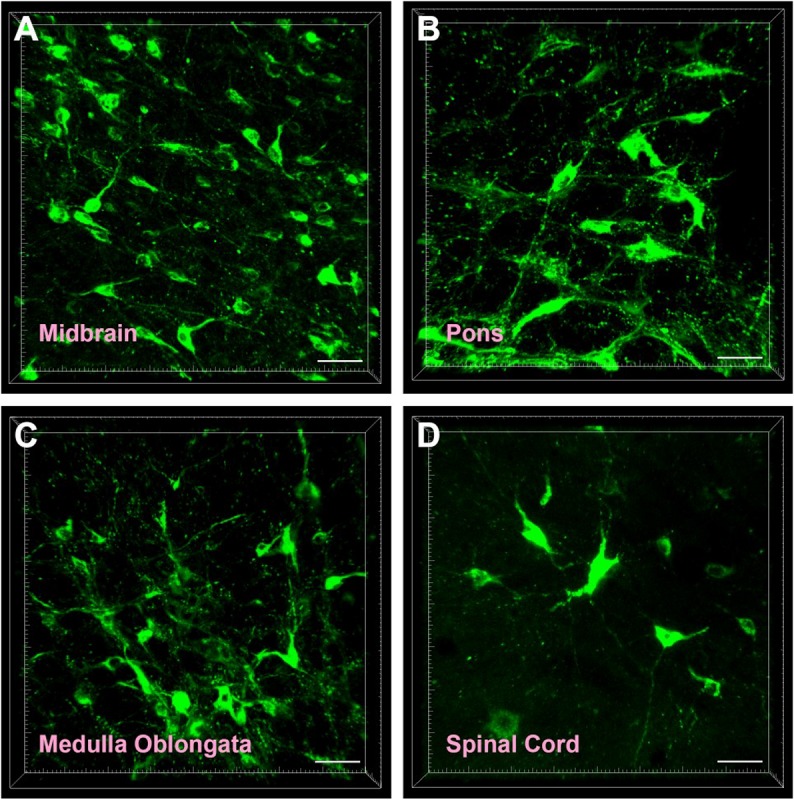
Cellular morphology of CB-positive neurons. Representative photographs from the midbrain **(A)**, pons **(B)**, medulla oblongata **(C)**, and spinal cord **(D)**. Scale bar = 40 μm.

The CB-positive neurons were round or fusiform in shape. Sizes of these neurons are different, and the processes are obvious (Figure 2A). In the reticular structure of the brainstem, the neurons are large and appear in polygonal shape; they have many processes, and the processes have many branches (Figures 2B,C). In the spinal cord, the neurons are fusiform, and the processes are obvious and longer (Figure 2D).

### Connection Sites of the Brainstem and Spinal Cord

The brainstem and spinal cord projections to the CSF-contacting nucleus can be identified by CB labeling.

**FIGURE 3:**
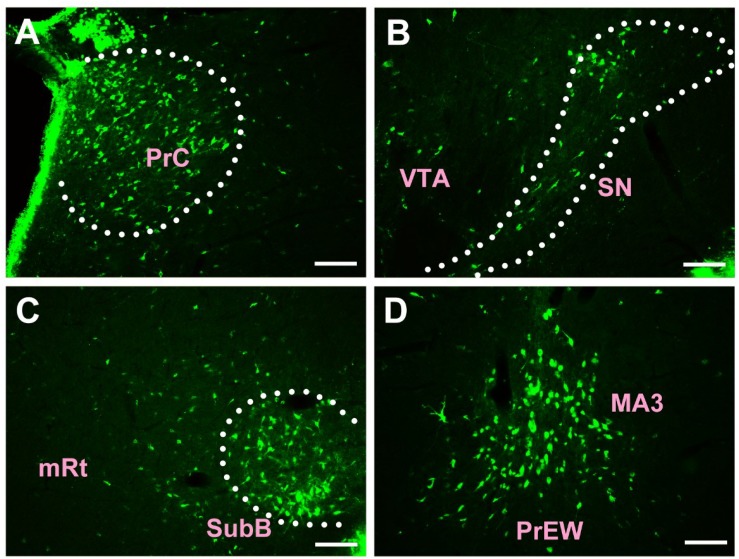
The distribution of neurons labeled by CB in the midbrain Part I **(A–D)**. PrC, precommissural nucleus; VTA, ventral tegmental area; SN, substantia nigra; mRt, mesencephalic reticular formation; SubB, subbrachial nucleus; MA3, medial accessory oculomotor nucleus; PrEW, pre-Edinger-Westphal nucleus. Scale bar = 100 μm.

**FIGURE 4:**
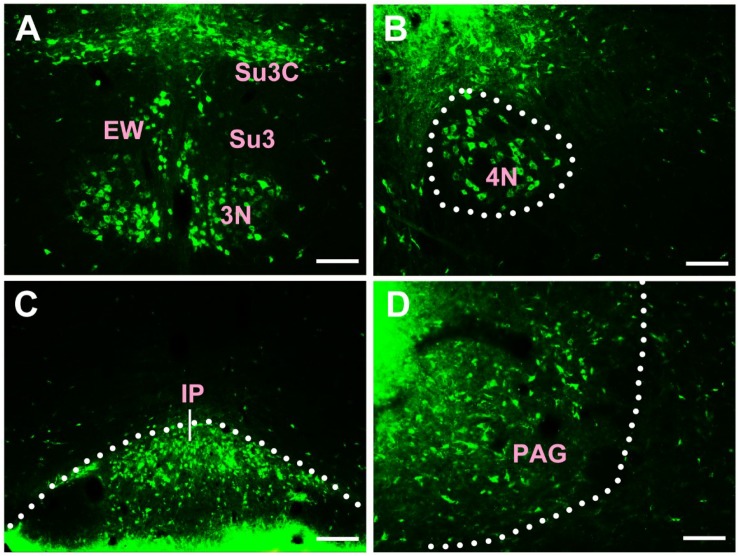
The distribution of neurons labeled by CB in the midbrain Part II **(A–D)**. EW, Edinger-Westphal nucleus; Su3, supraoculomotor periaqueductal gray; Su3C, supraoculomotor cap; 3N, oculomotor nucleus; 4N, trochlear nucleus; IP, interpeduncular nucleus; PAG, periaqueductal gray. Scale bar = 100 μm.

In the midbrain, CB-positive neuron can be found in the precommissural nucleus (PrC), periaqueductal gray (PAG), nucleus of Darkschewitsch (DK), medial accessory oculomotor nucleus (MA3), pre-Edinger-Westphal nucleus (PrEW), Edinger-Westphal nucleus (EW), rostral linear nucleus of the raphe (RLi), interfascicular nucleus (IF), interstitial nucleus of Cajal (InC), red nucleus (R), ventral tegmental area (VTA), substantia nigra (SN), medial pretectal nucleus (MPT), posterior pretectal nucleus (PPT), olivary pretectal nucleus (OPT), nucleus of the optic tract (OT), posterior thalamic nuclear group (Po), interpeduncular nucleus (IP), supraoculomotor cap (Su3C), supraoculomotor periaqueductal gray (Su3), oculomotor nucleus (3N), trochlear nucleus (4N), mesencephalic reticular formation (mRt), superior colliculus (SC), inferior colliculus (IC), caudal linear nucleus of the raphe (CLi), subbrachial nucleus (SubB), and retrorubral field (RRF) (Figures 3, 4).

Among them, the PrC, PAG, MA3, IP, Su3C, Su3, 3N, 4N, and SubB have dense projections; the PrEW, EW, IF, SN, MPT, PPT, OPT, OT, and mRt send moderate projections; and the DK, RLi, InC, R, VTA, Po, SC, IC, CLi, and RRF send sparse projections to the CSF-contacting nucleus (Figures 3, 4).

**FIGURE 5:**
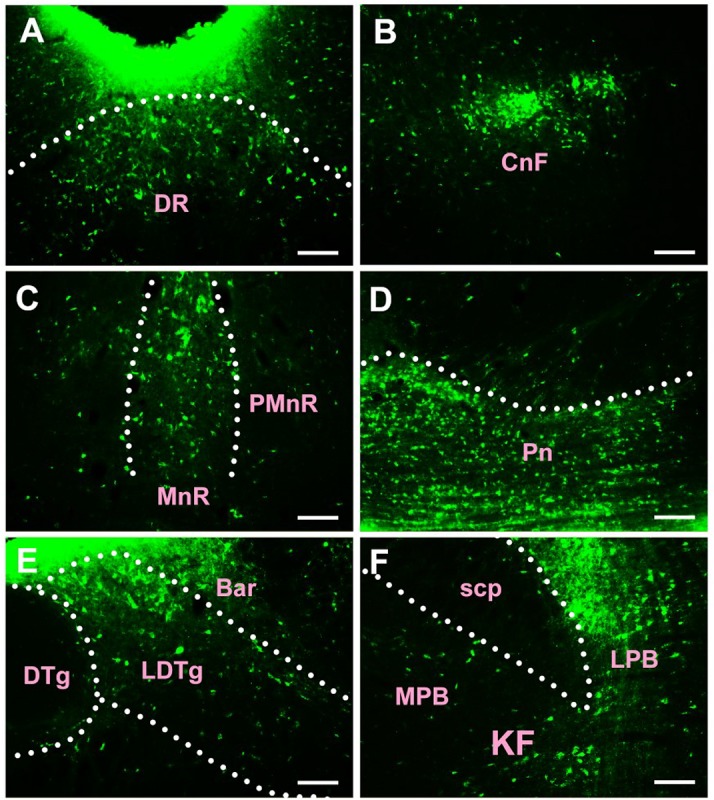
The distribution of neurons labeled by CB in the pons **(A–F)**. DR, dorsal raphe nucleus; CnF, cuneiform nucleus; MnR, median raphe nucleus; PMnR, paramedian raphe nucleus; Pn, pontine nuclei; DTg, dorsal tegmental nucleus; LDTg, laterodorsal tegmental nucleus; Bar, Barrington’s nucleus; scp, superior cerebellar peduncle; LPB, lateral parabrachial nucleus; MPB, medial parabrachial nucleus; KF, Kolliker Fuse nucleus. Scale bar = 100 μm.

In the pons, CB-positive neuron can be found in the pontine nuclei (Pn), dorsal raphe nucleus (DR), median raphe nucleus (MnR), paramedian raphe nucleus (PMnR), isthmic reticular formation (isRt), pedunculopontine tegmental nucleus (PTg), subpeduncular tegmental nucleus (SPTg), microcellular tegmental nucleus (MiTg), precuneiform area (PrCnF), cuneiform nucleus (CnF), laterodorsal tegmental nucleus (LDTg), medial paralemniscial nucleus (MPL), nucleus of the lateral lemniscus (LL), A7 noradrenaline cells (A7), central gray (CG), pontine reticular nucleus oral part (PnO), pontine reticular nucleus caudal part (PnC), pontine reticular nucleus ventral part (PnV), Barrington’s nucleus (Bar), subcoeruleus nucleus (SubC), lateral parabrachial nucleus (LPB), medial parabrachial nucleus (MPB), Kolliker Fuse nucleus (KF), supratrigeminal nucleus (Su5), motor nucleus of trigeminal nerve (Mo5), medioventral periolivary nucleus (MVPO), lateroventral periolivary nucleus (LVPO), locus coeruleus (LC), superior vestibular nucleus (SuVe), medial vestibular nucleus parvicellular part (MVePC), medial vestibular nucleus magnocellular part (MVeMC), lateral vestibular nucleus (LVe), spinal vestibular nucleus (SpVe), nucleus of origin of efferents of the vestibular nerve (EVe), supragenual nucleus (SGe), ventral cochlear nucleus (VC), dorsal cochlear nucleus (DC), lateral superior olive (LSO), superior paraolivary nucleus (SPO), nucleus of the trapezoid body (Tz), abducens nucleus (6N), paraabducens nucleus (Pa6), raphe interpositus nucleus (RIP), raphe pallidus nucleus (RPa), dorsal paragigantocellular nucleus (DPGi), gigantocellular reticular nucleus (Gi), gigantocellular reticular nucleus alpha part (GiA), gigantocellular reticular nucleus ventral part (GiV), raphe magnus nucleus (RMg), facial nucleus (7N), A5 noradrenaline cells (A5), intermediate reticular nucleus alpha part (IRtA), parvicellular reticular nucleus alpha part (PCRtA), parapyramidal nucleus (PPy), lateral paragigantocellular nucleus alpha part (LPGiA), and lateral paragigantocellular nucleus external part (LPGiE) (Figure 5).

Among them, the Pn, DR, MnR, CnF, LDTg, CG, Bar, LPB, KF, LC, MVePC, EVe, SGe, DC, and 6N have strong projections; the PMnR, PTg, MPB, MVPO, SuVe, MVeMC, VC, TZ, RPa, RMg, LPGiA, and LPGiE have moderate projections; and the isRt, SPTg, MiTg, PrCnF, MPL, LL, A7, PnO, PnC, PnV, SubC, Su5, Mo5, LVPO, LVe, LSO, SPO, Pa6, RIP, DPGi, Gi, GiA, GiV, 7N, A5, IRtA, PCRtA, and PPy have sparse projections to the CSF-contacting nucleus (Figure 5).

**FIGURE 6:**
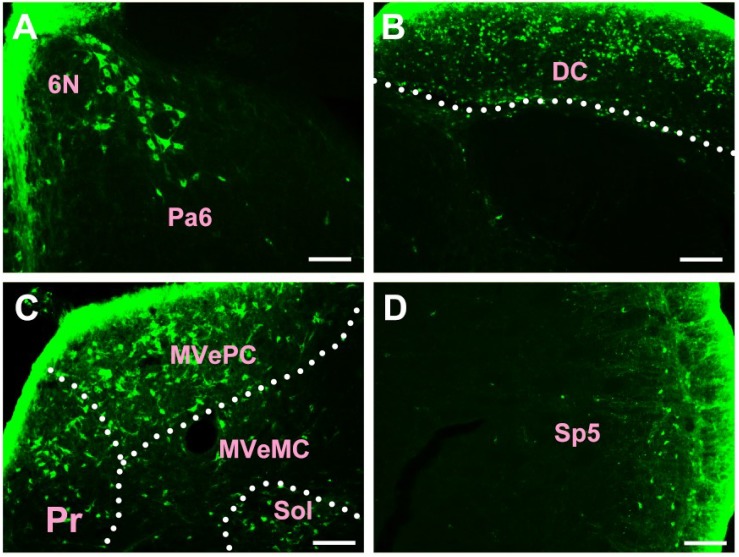
The distribution of neurons labeled by CB in the medulla oblongata Part I **(A–D)**. 6N, abducens nucleus; Pa6, paraabducens nucleus; DC, dorsal cochlear nucleus; Pr, prepositus nucleus; MVePC, medial vestibular nucleus parvicellular part; MVeMC, medial vestibular nucleus magnocellular part; Sol, solitary nucleus; Sp5, spinal trigeminal nucleus. Scale bar = 100 μm.

**FIGURE 7:**
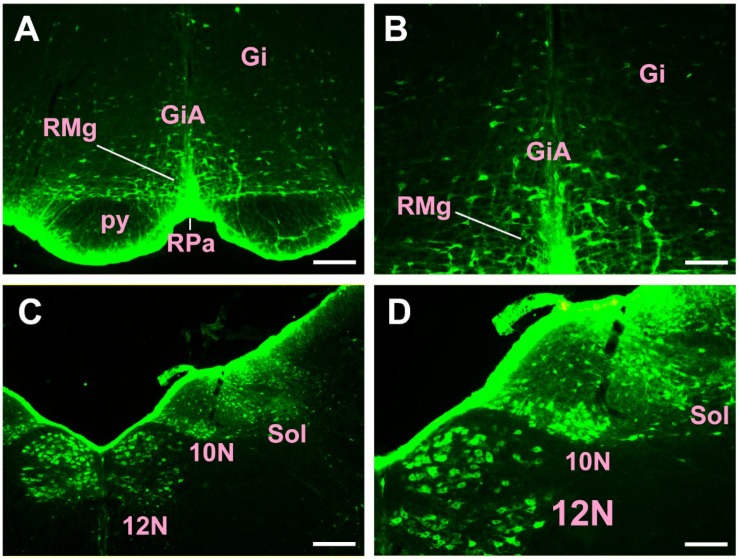
The distribution of neurons labeled by CB in the medulla oblongata Part II **(A–D)**. Gi, gigantocellular reticular nucleus; GiA, gigantocellular reticular nucleus alpha part; RMg, raphe magnus nucleus; RPa, raphe pallidus nucleus; py, pyramidal tract; 10N, dorsal motor nucleus of vagus; 12N, hypoglossal nucleus; Sol, solitary nucleus. Scale bar = 100 μm.

**FIGURE 8:**
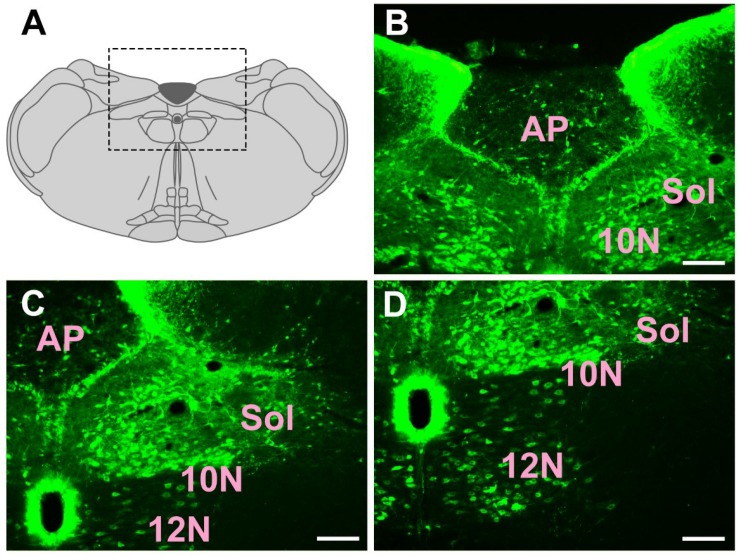
The distribution of neurons labeled by CB in the medulla oblongata Part III **(A–D)**. 10N, dorsal motor nucleus of vagus; 12N, hypoglossal nucleus; Sol, solitary nucleus; AP, area postrema. Scale bar = 100 μm.

**FIGURE 9:**
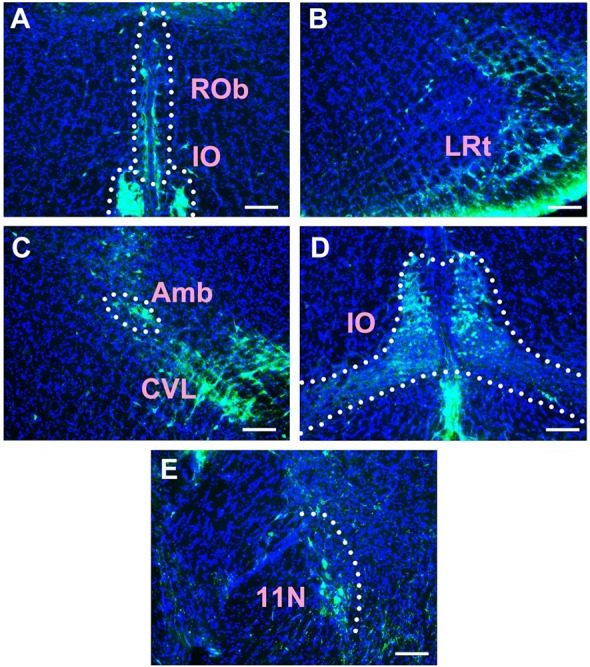
The distribution of neurons labeled by CB in the medulla oblongata Part IV **(A–E)**. Rob, raphe obscurus nucleus; IO, inferior olive; LRt, lateral reticular nucleus; Amb, ambiguus nucleus; CVL, caudoventrolateral reticular nucleus; 11N, accessory nerve nucleus. Scale bar = 100 μm.

**FIGURE 10:**
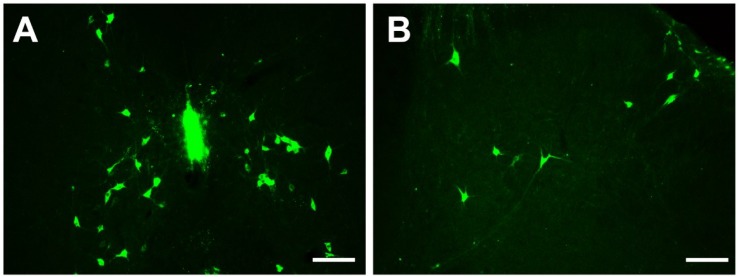
Distribution of neurons labeled by CB in the spinal cord **(A,B)**. Scale bar = 100 μm.

In the medulla oblongata and spinal cord, CB-positive neurons can be found in the prepositus nucleus (Pr), solitary nucleus (Sol), raphe obscurus nucleus (Rob), epifascicular nucleus (EF), rostroventrolateral reticular nucleus (RVL), caudoventrolateral reticular nucleus (CVL), spinal trigeminal nucleus (Sp5), ambiguus nucleus (Amb), Botzinger complex (Bo), nucleus of Roller (Ro), external cuneate nucleus (ECu), cuneate nucleus (Cu), inferior olive (IO), dorsal motor nucleus of vagus (10N), hypoglossal nucleus (12N), rostral ventral respiratory group (RVRG), paratrigeminal nucleus (Pa5), lateral reticular nucleus (LRt), gracile nucleus (Gr), area postrema (AP), A1 noradrenaline cells (A1), A2 noradrenaline cells (A2), accessory nerve nucleus (11N), intermediate reticular nucleus (IRt), parvicellular reticular nucleus (PCRt), medullary reticular nucleus dorsal part (MdD), medullary reticular nucleus ventral part (MdV), and spinal cord (Figures 6–10).

Among them, the Pr, Sol, RVL, CVL, Amb, 10N, 12N, AP, and A1 have strong projections; the Rob, Sp5, Bo, IO, LRt, A2, 11N, and IRt have moderate projections; and EF, Ro, ECu, Cu, RVRG, Pa5, Gr, PCRt, MdD, MdV, and spinal cord have sparse projections to the CSF-contacting nucleus (Figures 6–10).

**FIGURE 11:**
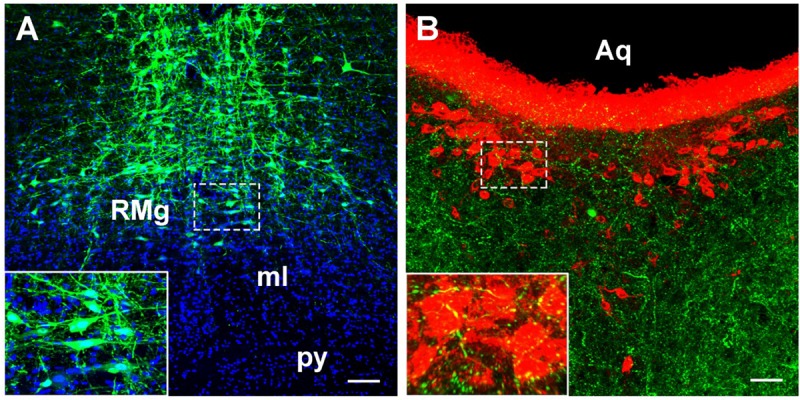
The verification of the RMg projection to the CSF-contacting nucleus by anterograde tracing. **(A)** The injection site of the anterograde tracer rAAV2/9-hSyn-EGFP into the RMg. **(B)** The axon terminals of the RMg (green) make close appositions with the neurons in the CSF-contacting nucleus (red). RMg, raphe magnus nucleus; py, pyramidal tract; ml, medial lemniscus; Aq, aqueduct. Scale bar = 100 μm in **A**; Scale bar = 50 μm in **B**.

In summary, CB-positive neurons were distributed in 4 functional areas including 112 sub-regions in the brainstem and spinal cord. However, their distribution ranged from sparse to dense in each functional region. Other parts did not contain CB-positive neurons. In addition, the retrograde results were further verified by the anterograde method. After the anterograde tracer rAAV2/9 injection into the RMg, large number of axon terminals can be detected in the CSF-contacting nucleus (Figure 11).

### 3D Reconstruction of the CB-Positive Neurons From the Brainstem and Spinal Cord

**FIGURE 12:**
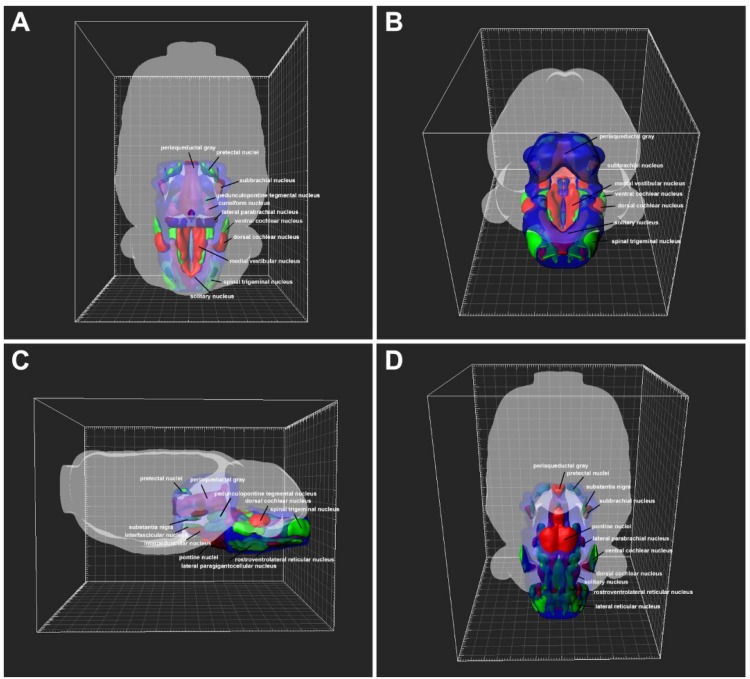
3D view of the patterns of the brainstem and spinal cord connections to the CSF-contacting nucleus. **(A)** Dorsal view, **(B)** posterior view, **(C)** lateral view, **(D)** ventral view. The red areas indicate strong connections; green areas indicate moderate connections; and blue areas indicate weak connections to the CSF-contacting nucleus.

The distributions of CB-positive neurons throughout the brainstem and spinal cord were three dimensionally reconstructed. The densities of the connections were obvious in the 3D view. The red areas indicated dense connections (PrC, PAG, MA3, IP, Su3C, Su3, 3N, 4M, SubB, Pn, DR, MnR, CnF, LDTg, CG, Bar, LPB, KF, LC, MVePC, EVe, SGe, DC, 6N, Pr, Sol, RVL, CVL, Amb, 10N, 12N, AP, and A1); green areas indicated moderate connections (PrEW, EW, IF, SN, MPT, PPT, OPT, OT, mRt, PMnR, PTg, MPB, MVPO, SuVe, MVeMC, VC, Tz, RPa, RMg, LPGiA, LPGiE, ROb, Sp5, Bo, IO, LRt, A2, 11N, and IRt); and blue areas indicated sparse connections (DK, RLi, InC, R, VTA, Po, SC, IC, CLi, RRF, isRt, SPTg, MiTg, MPL, LL, A7, PnO, PnC, PnV, SubC, Su5, Mo5, LVPO, LVe, SpVe, LSO, SPO, Pa6, RIP, DPGi, Gi, GiA, GiV, 7N, A5, IRtA, PCRtA, PPy, EF, Ro, ECu, Cu, RVRG, Pa5, Gr, PCRt, MdD, MdV, and spinal cord) to the CSF-contacting nucleus (Figure 12).

### The Amount of Projection From the Brainstem and Spinal Cord to the CSF-Contacting Nucleus

**FIGURE 13:**
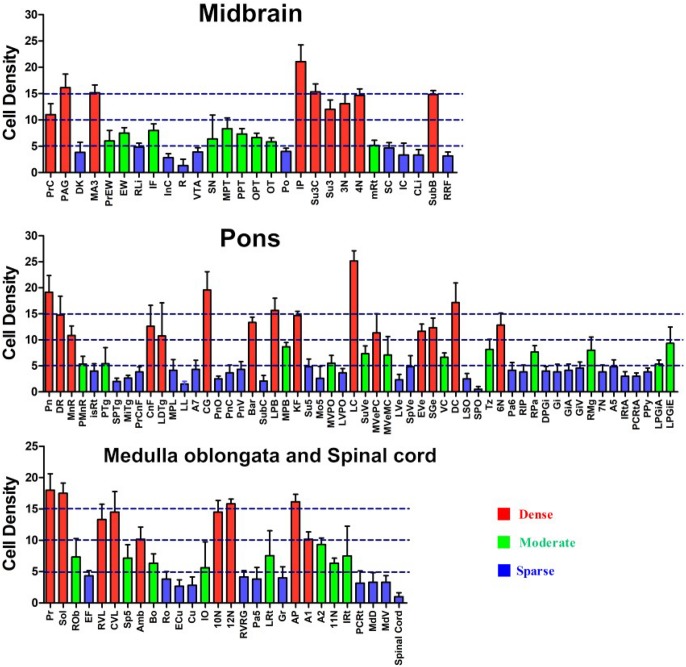
Whole brainstem and spinal cord statistics of CB-positive cell input to the CSF-contacting nucleus (mean ± SD, *n* = 6).

In the brainstem and spinal cord, the CB-positive neurons were found in 112 sub-regions. The amount of projection from these regions to the CSF-contacting nucleus is shown in Figure 13.

## Discussion

The CSF-contacting nucleus is a unique nucleus in the brain. It has non-synaptic connections with blood vessels and CSF via the CSF-contacting neurons; this connection plays an important role in the regulation of body fluids. Additionally, it has synaptic connections with non-CSF-contacting neurons via the CSF-contacting neurons; this connection facilitates neuron-neuron crosstalk in the brain. The unique anatomical feature of the CSF-contacting nucleus is that it may be a key structure bridging the nervous and humoral regulating systems. It has been uncovered that this nucleus receives the projection from central nervous system, although the bidirectional synapses between CSF-contacting and non-CSF-contacting neurons were observed in the parenchyma by electron microscopy (Liang et al., 2007). Herein, we include a systematic discussion of the projections from different functional zones in the brainstem and spinal cord to the CSF-contacting nucleus. As the fibers of passage may have been taken up by the retrograde tracer CB, we also verify the results by the anterograde method.

**FIGURE 14:**
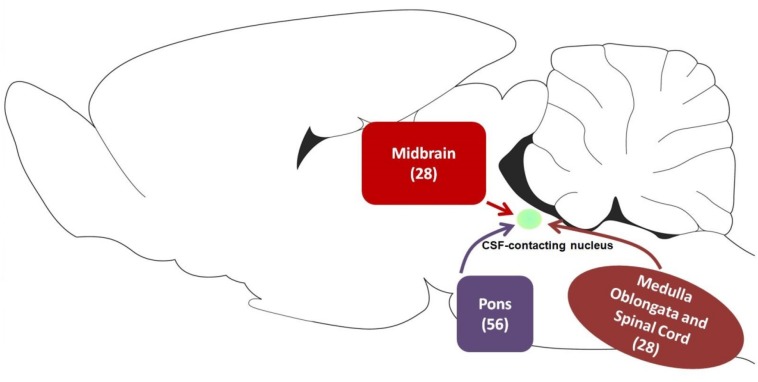
The schematic diagram of projections from functional areas in the brainstem and spinal cord to the CSF-contacting nucleus. Among them, midbrain contains 28 sub-regions, pons contains 56 sub-regions, and medulla oblongata and spinal cord contain 28 sub-regions.

Our results indicate that the CSF-contacting nucleus received extensive projections from 4 functional areas including 112 sub-regions of the brainstem and spinal cord (Figure 14). Among them, the CSF-contacting nucleus receives the connections from the brainstem cranial nerve nuclei, such as trigeminal nuclei and solitary nucleus. The CSF-contacting nucleus is also located in the brainstem, but the processes stretch into the CSF. The nucleus is more sensitive to the peripheral tracer CB-HRP or CB which implies that the CSF-contacting nucleus has the characteristics of the cranial nerve nuclei. According to the connection patterns of the CSF-contacting nucleus with the functional areas of the brainstem and spinal cord, the biological functions of the CSF-contacting nucleus can be predicted. Whilst the function of the CSF-contacting nucleus is not known, the following are the functional implications of these connections.

### Pain

The pain descending inhibitory system is located in the brainstem. This system can send fibers in a top-down manner to the dorsal spinal horn to inhibit pain transmission (Millan, 2002). The system comprises of the PAG, DR, rostral-ventromedial medulla (RVM), and LC (Reynolds, 1969; Francois et al., 2017; Torralba et al., 2018; Zhang et al., 2018). The system also sends dense connections to the CSF-contacting nucleus, which might be a novel pathway in pain modulation.

Apart from the descending inhibitory system, other regions (CnF, PTg, A5, A7, LRt, MdD, and spinal cord) (Zemlan and Behbehani, 1988; Dugast et al., 2003; Mravec et al., 2012; Huma et al., 2015; Francois et al., 2017; de Oliveira et al., 2018) can also regulate nociception and send projections to the CSF-contacting nucleus. For example, deep brain stimulation of the CnF neurons is known to produce antinociceptive effect (Zemlan and Behbehani, 1988). Lesion in the PTg impair the antinociceptive effects (de Oliveira et al., 2018).

### Visceral Activity

In the brainstem, the Bar, CnF, Gi, LPB, MPB, PTg, 10N, A1, Amb, Bo, RVL, RVRG, and Sol are important for visceral activity modulation; they have extensive connections with the CSF-contacting nucleus. The Bar engages in modulating the bladder functions (Blanco et al., 2014). Manipulation of the neuronal functions in the CnF, Gi, PTg, A1, Amb, and RVL has great effects on cardiovascular responses (Chan and Chan, 1983; Elliott et al., 1985; Balan Junior et al., 2004; Topchiy et al., 2010; Shafei et al., 2012; Brailoiu et al., 2017). The Bo and RVRG are responsible for respiratory control. The Bo has been described to be involved in the compensatory responses to hypoxia (Alberto et al., 2014), while neurons of the RVRG can directly project to the phrenic motor neurons and discharge an inspiratory pattern (Hayakawa et al., 2004).

The LPB and MPB are located around the superior cerebellar peduncle (scp). They can receive various visceral inputs from the Sol and spinal cord and can relay the visceral information to other regions of the brain (Palmiter, 2018). The Sol is one of the most important sites involved in visceral modulation, and it is regarded as the entry point of afferent signals from various visceral mechanosensors, chemosensors, and C-fibers (Kotmanova et al., 2018; You et al., 2018). The 10N can integrate information and control the visceral activities (Browning et al., 2005; Cruz et al., 2007).

### Sleep and Arousal

The CSF-contacting nucleus receives broad projections along the brainstem reticular formation—a region of the brain that plays a key role in regulating sleep and arousal (Moruzzi and Magoun, 1949). The PnO is a part of the ascending reticular activating system, and it regulates rapid eye movement sleep (REMS) and wakefulness (Nunez et al., 2010; Silvia et al., 2013; Vanini and Baghdoyan, 2013). The Gi has projections to the midbrain reticular formation and intralaminar thalamic nuclei, which help modulate arousal (Martin et al., 2011). During the REMS, activity of the LRt neurons changes significantly (Stettner et al., 2013).

Apart from the brainstem reticular formation, other regions of the brain that innervate the CSF-contacting nucleus, such as the DR and LC, can also modulate sleep and arousal. Pharmacological intervention the DR can significantly change REMS (Monti and Jantos, 2018). The LC is a key arousal node, and it is pivotal for modulating consciousness and effects of general anesthetics (Du et al., 2018).

### Emotion

The CSF-contacting nucleus receives projections from the EW, IP, PAG, DR, LC, and MnR, which might be associated with emotion. The EW is a region implicated in stress responses and different aspects of stress-related behaviors (Xu et al., 2010, 2014). Lesion-based studies demonstrated that the IP is involved in regulating anxiety and fear (Mclaughlin et al., 2017). The PAG is regarded as an integrated site for coordinating rapid responses to aversive stimuli, such as those provoking panic, anxiety, or fear (Lowery-Gionta et al., 2018). The DR and MnR contain the major serotonergic (5-HT) populations (Pollak Dorocic et al., 2014). Both of the nuclei and 5-HT are involved in anxiety and depression, and they are crucial and central drug targets (Lanzenberger et al., 2012; An et al., 2013, 2016; Lopez Hill et al., 2013; Campos et al., 2018; de Souza et al., 2018; Donner et al., 2018). The LC is the major noradrenergic system with regard to stress and major depressive disorder (Chandley and Ordway, 2012; Fan et al., 2018).

### Drug Addiction

The CSF-contacting nucleus receives input from the EW, IP, LDTg, and LPGi, which might be associated with drug addiction. After oral self-administration of ethanol, the EW shows significantly elevated c-Fos expression (Bachtell et al., 1999; Giardino et al., 2011). Pharmacological studies show that the IP mediates behavioral responses to nicotine reinforcement, aversion, and withdrawal (Ramiro et al., 2009; Antolin-Fontes et al., 2015; Morton et al., 2018). The LDTg is involved in drug reward processing and reinforcement in the development of addiction and abuse (Kaneda, 2018). The LPGi has been shown to play an important critical role in the development of opiate tolerance and dependence (Ahmadi-Soleimani et al., 2017).

In this study, we used tract-tracing method to reveal the CSF-contacting nucleus input patterns from the brainstem and spinal cord. The unique morphological feature of the CSF-contacting nucleus is that the somata are located in the brain parenchyma, which can receive inputs from the above-mentioned areas; the processes can form synaptic and non-synaptic connections with non-CSF-contacting neurons, CSF, or plasma. For one aspect, it might form the brainstem and spinal cord→CSF-contacting nucleus→non-CSF-contacting neurons circuits and participate in the regulation of life activities via neuron-neuron crosstalk. For another aspect, it might be involved in the brainstem and spinal cord→CSF-contacting nucleus→CSF/plasma circuit and modulate physiological functions via neuron-fluid interactions. According to the connection regularities of CSF-contacting nucleus from the brainstem and spinal cord, we preliminary conclude that the CSF-contacting nucleus involved in modulation pain, visceral activity, sleep and arousal, emotion, and drug addiction, etc. The current study provides morphological evidence for unveiling the significance of the CSF-contacting nucleus.

## Data Availability Statement

The data that support the findings of this study are available from the corresponding author, upon reasonable request.

## Ethics Statement

All animal experiments were approved by and performed in accordance with the guidelines of the Committee for Ethical Use of Laboratory Animals of Xuzhou Medical University.

## Author Contributions

S-YS and L-CZ designed the study and prepared the manuscript. S-YS, YL, X-MZ, Y-HL, C-YB, C-JS, JH, and J-LC conducted the studies. All authors read and approved the manuscript.

## Conflict of Interest

The authors declare that the research was conducted in the absence of any commercial or financial relationships that could be construed as a potential conflict of interest.
